# Candesartan restores pressure-induced vasodilation and prevents skin pressure ulcer formation in diabetic mice

**DOI:** 10.1186/s12933-015-0185-4

**Published:** 2015-02-18

**Authors:** Aurore Danigo, Mohamad Nasser, Flavien Bessaguet, James Javellaud, Nicole Oudart, Jean-Michel Achard, Claire Demiot

**Affiliations:** EA6309, School of Medecine and Pharmacy, University of Limoges, 87025 Limoges Cedex, France; EA3842, School of Medecine and Pharmacy, University of Limoges, 87025 Limoges Cedex, France

**Keywords:** Candesartan, Pressure-induced vasodilation, Streptozotocin, Pressure ulcer

## Abstract

**Background:**

Angiotensin II type 1 receptor (AT1R) blockers have beneficial effects on neurovascular complications in diabetes and in organ’s protection against ischemic episodes. The present study examines whether the AT1R blocker candesartan (1) has a beneficial effect on diabetes-induced alteration of pressure-induced vasodilation (PIV, a cutaneous physiological neurovascular mechanism which could delay the occurrence of tissue ischemia), and (2) could be protective against skin pressure ulcer formation.

**Methods:**

Male Swiss mice aged 5–6 weeks were randomly assigned to four experimental groups. In two groups, diabetes was induced by a single intraperitoneal injection of streptozotocin (STZ, 200 mg.kg^−1^). After 6 weeks, control and STZ mice received either no treatment or candesartan (1 mg/kg-daily in drinking water) during 2 weeks. At the end of treatment (8 weeks of diabetes duration), C-fiber mediated nociception threshold, endothelium-dependent vasodilation and PIV were assessed. Pressure ulcers (PUs) were then induced by pinching the dorsal skin between two magnetic plates for three hours. Skin ulcer area development was assessed during three days, and histological examination of the depth of the skin lesion was performed at day three.

**Results:**

After 8 weeks of diabetes, the skin neurovascular functions (C-fiber nociception, endothelium-dependent vasodilation and PIV) were markedly altered in STZ-treated mice, but were fully restored by treatment with candesartan. Whereas in diabetes mice exposure of the skin to pressure induced wide and deep necrotic lesions, treatment with candersartan restored their ability to resist to pressure-induced ulceration as efficiently as the control mice.

**Conclusion:**

Candesartan decreases the vulnerability to pressure-induced ulceration and restores skin neurovascular functions in mice with STZ-induced established diabetes.

## Background

Diabetes is a particularly important risk factor for the development of chronic wounds because it is often associated with vasculopathy and neuropathy. Diabetic neuropathy (DN) is a common complication of diabetes mellitus (DM). Many of diabetic patients develop a DN, most commonly seen as distal symmetrical sensorimotor polyneuropathy [1]. Diabetic patients who express DN tend to develop spontaneous skin injury as diabetic foot ulcer. The common understanding is that sensory nerve impairment diminishes the perception of pain that is protective when tissue injury occurs. Patients are not aware of pain, and lose protective withdrawal reflex to avoid tissue harm. While this certainly remains true, we recently reported that, for the same imposed unavoidable pressure stimulus, diabetic mice develop more severe skin ulceration than healthy control mice, indicating that in diabetic mice, intrinsic physiological mechanisms of skin adaptative protection against ischemic pressure are impaired [2].

Pressure-induced vasodilation (PIV) was reported as a physiological neurovascular response to local application of low pressure to the skin that increases cutaneous blood flow, thereby delaying the occurrence of tissue ischemia, and thus protecting the skin against pressure [3,4]. The cutaneous neuronal mechanosensor initiating the vasodilatory response has recently been identified as the Acid-sensing ion channel 3 (Asic3), a mechanosensitive channel [5]. The succession of steps leading to PIV involves pressure perception in nociceptive sensory small fibers neurons mediated by Asic3, resulting in release of the vasodilator calcitonin gene-related peptide (CGRP) from afferent sensory nerve endings. CGRP then triggers the liberation from the endothelium of nitric oxide (NO) that is responsible for vascular smooth muscle cell relaxation. Hence, a normal PIV response requires both the functional integrity of small fibers sensory neurons and endothelium-dependent relaxation, which are both altered by diabetes. Indeed, PIV is impaired in experimental models of diabetes in mice [6], as well as in diabetes patients [7], reflecting the vulnerability of diabetic skin to pressure.

Overwhelming evidence has accumulated showing that activation of the tissue renin-angiotensin system (RAS) is involved in the alteration of endothelium-dependent relaxation in diabetes and that blockade of the RAS has beneficial effects on endothelial function. Successful clinical trials have established opposing the RAS, with either ACE (Angiotensin-converting enzyme) inhibitors (ACEIs) or angiotensin-receptor blockers (ARBs), as first line treatment not only for hypertension, but also for prevention of cardiovascular disease, nephropathy and retinopathy in diabetic patients [8].

Although more scarcely studied, experimental animal studies also suggest a beneficial effect of RAS blockade on diabetic neuropathy. ACEIs or ARBs improve neural functions in streptozotocin-diabetes rats [9-12] and the ARB olmesartan has been shown to improve peripheral nerve dysfunction in Zucker diabetic fatty rats [13]. Clinical data are very limited, but a double-blind controlled trial conducted in 41 normotensive type I or II diabetes patients with mild neuropathy suggested a beneficial effect of the ACEI trandolapril on peripheral neuropathy [14]. The double protective action of RAS blockade towards diabetes induced endothelial dysfunction and peripheral neuropathy thus raised the hypothesis that it might be well suited for preventing diabetes induced PIV impairment, allowing the skin to resist to pressure-induced ischemia. The present study was thus designed to determine whether the ARB candesartan, administered in long-term streptozotocin-diabetic mice with established neurovascular dysfunction, could restore PIV and prevent pressure-induced skin ulcer formation.

## Methods

### Experimental groups of treatment

**Figure 1:**
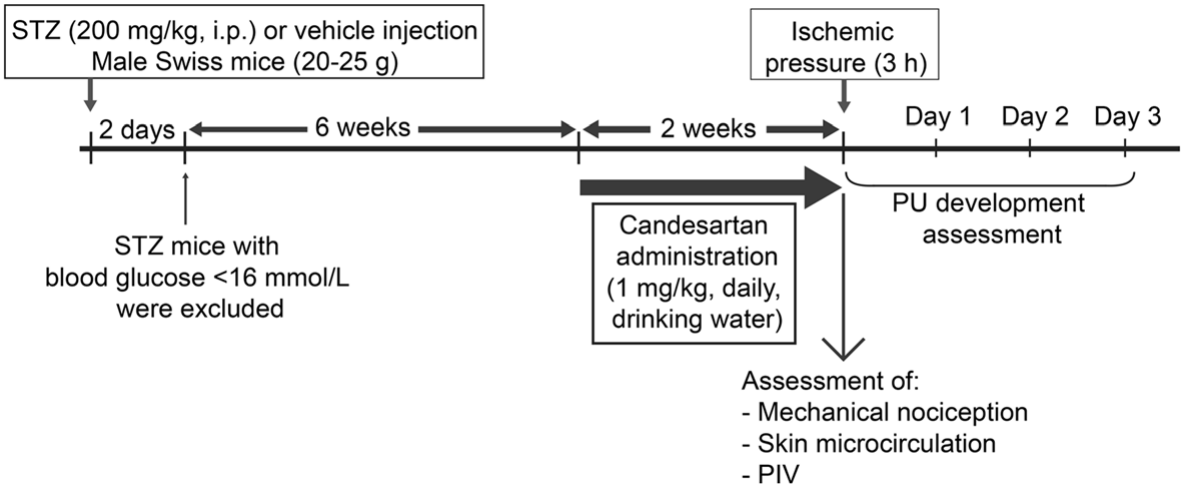
**Schematic representation of study design.** PU: pressure ulcer, STZ: streptozotocin.

Male Swiss mice aged 5–6 weeks (20-25 g) were obtained from Depré (Saint Doulchard, France), and maintained on a 12 h light/dark cycle with food and water available ad libitum*.* Mice were randomly assigned to 4 experimental groups (n = 80, so 20 per group). In two groups, diabetes was induced by a single intraperitoneal injection of streptozotocin (STZ) (200 mg.kg^−1^; Sigma-Aldrich, Lyon, France), whereas in the two control groups the mice received an intraperitoneal injection of the vehicle (citrate buffer, pH 4.5). In the diabetic groups hyperglycemia occurred 2 days after STZ injection and was verified using Accu-Check Active glucometer (Roche, Lyon, France). Mice were excluded when blood glucose was < 288 mg.dl^−1^ two days after injection. After 6 weeks of diabetes duration, one control and one STZ group were left untreated, while the other control group and the other STZ group were treated with candesartan (Astra-Zeneca, 1 mg.kg^−1^ per day in drinking water) for two additional weeks (Figure 1). The concentrations of candesartan in drinking water were adapted according to the daily water quantity absorbed by the mice in order to insure each mouse received approximately 1 mg.kg^−1^ per day.

Assessments of mechanical nociception, skin microcirculation reactivity and cutaneous neurovascular functions, and induction of ulcer formation by pressure, were then performed after 8 weeks of diabetes. The stability of cutaneous temperature and systolic arterial blood pressure were controlled throughout the experiments of skin microcirculation reactivity and cutaneous neurovascular functions.

All experiments were carried out according to protocols approved by the Ethics Committee of Animal Experiments of Limousin (CREEAL-n°1-2013-2). The current investigation conformed to the guidelines for ethical care of experimental animals of the European Community and was approved by the French Agriculture Ministry (authorization n°87-019).

### Mechanical pressure algesia

Tail pressure thresholds were registered with the Paw/Tail Pressure Analgesia meter for the Randall-Selitto test (Bioseb, Vitrolles, France). Pressure increasing at a linear rate of 16 g s^−1^, with a cut-off at 250 g to avoid tissue injury, was applied to the base of the tail. The applied tail pressure that evoked biting and licking behaviour was registered and expressed in grams. Three tests separated by at least 15 minutes were performed for each animal.

### Skin microvascular function

Hair of the animals was removed 2 days before experiments, with a depilatory lotion to obtain a hairless area for skin Laser Doppler Flowmetry (LDF) measurements, local pressure application, and iontophoretic delivery. For experiments, animals were anesthetized with thiopental sodium (65 mg.kg^−1^intraperitoneal) and then placed in an incubator (Mediprema, Tours, France) warmed to maintain a stable cutaneous temperature (35.0 ± 0.5°C). Non-invasive tail blood pressure (Bionic Instruments, Tokyo, Japan) was recorded before and after experiments to verify systolic arterial blood pressure (SABP) stability.

### Skin microcirculation reactivity: Endothelium-independent and –dependent responses

Skin blood flow was recorded, using a laser Doppler multifiber probe (481–1; Permied, Stockholm, Sweden) during transcutaneous iontophoresis applied to a 1.2 cm^2^ area on the hairless back of animals. Skin blood was recorded at baseline, and during anodal acetylcholine (Ach) iontophoretic delivery (endothelium-dependent vasodilation assessment) or cathodal sodium nitroprusside (SNP) iontophoretic delivery (endothelium-independent vasodilation assessment) as previously described [15]. Vasodilator responses were reported as the maximal percentage increase from baseline in response to Ach orSNP.

### Skin neurovascular reactivity: Pressure-induced vasodilation

Skin blood flow in response to local pressure was measured by LDF, as previously described [3]. A laser Doppler probe (PF408, Periflux; Permied) was connected to a laser Doppler flowmeter (PF5000 Master, Periflux; Permied). The probe was placed in the middle of the hairless skull and external pressure was increased progressively at 2.2 Pa.s^−1^ through the probe. The LDF signal was digitized with 20 Hz sampling frequency, using a computerized acquisition system (Biopac, Santa Barbara, CA). Data collection started after a 1-min equilibration period before the onset of increasing pressure.

### Pressure ulcer model

Pressure ulcers were created on the dorsum of mice as described elsewhere [2,16]. The dorsal hair was shaved. After 24 h, the skin (epidermis, dermis and subcutaneous tissue layer, but not muscles) was pinched between two magnetic plates (10 mm diameter and 1 mm thick, with an average weight of 0.5 g and 10,000 Gauss magnetic force). This process created a compressive pressure of approximately 2,000 mmHg between the two magnets. Applying the magnets for 3 h had been shown to reproducibly elicit a skin ulcer in diabetic mice, but to only induce inconstant and minimal lesions in normal mice [2]. Skin ulcers develop progressively after withdrawal of the magnets, peaking at day three.

### Analysis of pressure ulcer formation

Each compressed area was photographed daily during three days using a 3.3 megapixel camera (Photo PC 3100Z; Epson, Nagano, Japan). Pressure ulcers were staged by visual assessment according the standardized ulcer scale [17]. Skin ulcer area percentage was calculated in the total compressed area using an image analyzer (Clara Vision, Orsay, France).

At day three tissue samples were dissected from the center of the lesion for a microscopic analysis. Samples were fixed overnight in buffered 4% formaldehyde solution, embedded in Finetek Tissu-Tek compound, and then frozen at −20°C. Sections of 12 μm were stained with hematoxylin and eosin. The samples were analyzed with an optical microscope (Leica).

Histological examination was based on histological modifications of the three skin layers and lack of tissue depth in the center of the sections (score 0: no histological modification of the three skin layers; score 1: three skin layers with disruption of fibers and histological epidermis modification; score 2: epidermal defect with necrotic areas in dermis; score 3: superficial dermal defect with necrotic regions; and score 4: deep dermal defect).

### Statistical analysis

Prism version 6.04 (GraphPad Software, Inc.; LaJolla, CA, USA) was used to make graphs and perform statistical tests. All data are presented as mean ± SE. A one-way analysis of variance (ANOVA) was used to evaluate differences among multiple groups with *p* values determined by the Newman-Keuls multiple range test with Gaussian distribution. A non-parametric Kruskal-Wallis test and Dunn’s multiple comparisons test were used for data which did not follow a Gaussian distribution. Differences were considered to be statistically significant at p < 0.05.

## Results

### Animals

Whereas after 8 weeks the control mice almost doubled their body weight, induction of diabetes markedly reduced the weight gain. Interestingly, candesartan had no effect on the blood glucose levels, although it did have an incomplete favourable effect on weight gains in the diabetic mice. Neither treatment with candesartan nor the diabetes induction significantly changed the systolic arterial blood pressure (Table 1).

**Table 1:** **Effects of diabetes and candesartan on weight gain, blood glucose and SABP**

**Groups**	**Control**	**Diabetic**	**Candesartan**	**Diabetic + Candesartan**
**Body weight gain (g)**	22.25 ± 0.86	4.94 ± 1.10***	19.25 ± 2.20	10.25 ± 1.36***
**Blood glucose (mg/dL)**	130.3 ± 7.94	466.7 ± 39.08	120.5 ± 5.09	461.4 ± 44.04
**SABP (mmHg)**	64.33 ± 3.38	73.58 ± 5.88	66.71 ± 4.93	64.92 ± 4.28

SABP: systolic arterial blood pressure. (n = 10 in each group, 1-way ANOVA followed by Newman-Keuls’s *post-hoc* test, ***P < 0.001: significance of the difference between candesartan-treated mouse group and respective untreated mouse group).

## Effects of candesartan on nociceptive, skin microcirculation and neurovascular function

### Assessment of mechanical algesia

**Figure 2:**
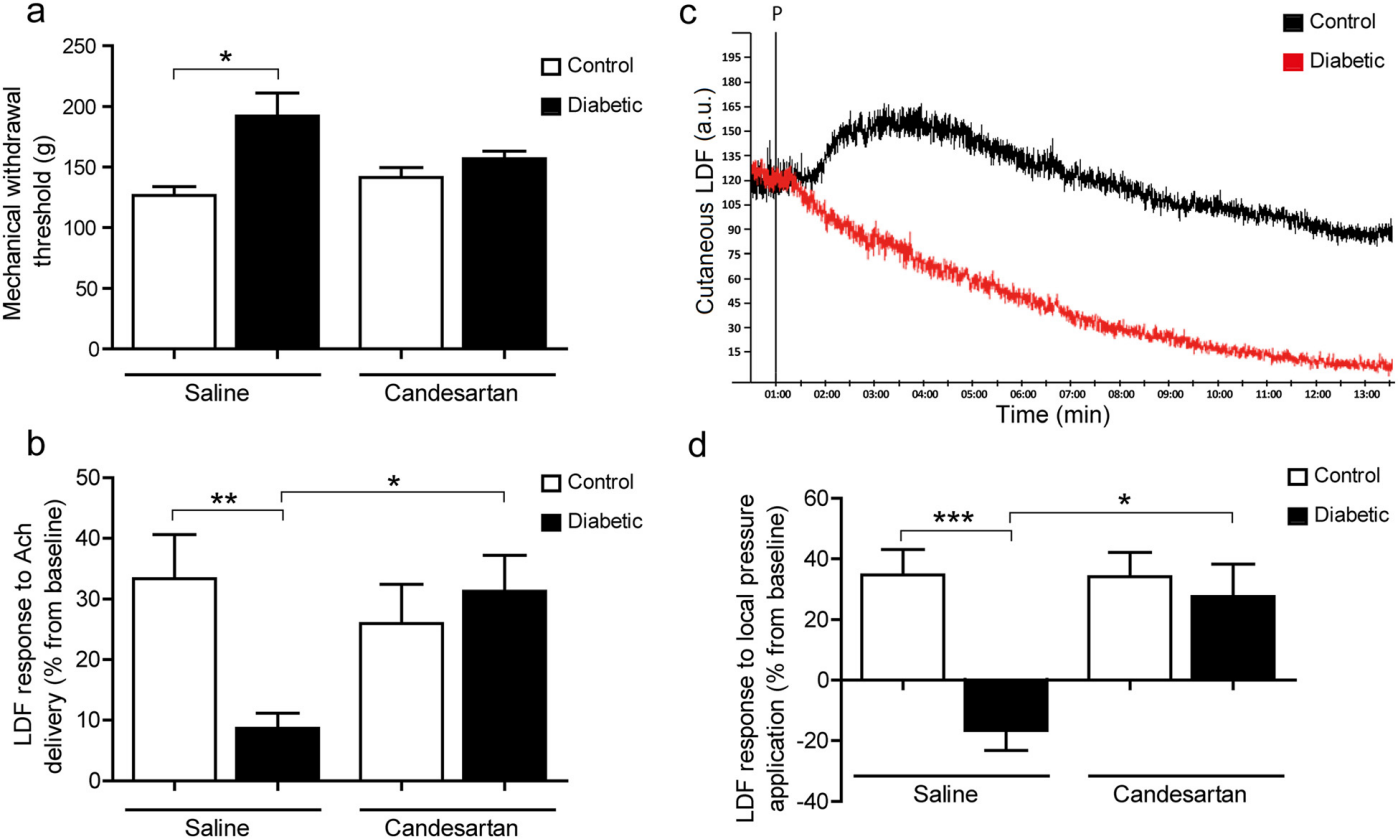
**Effects of candesartan on nociceptive, skin microcirculation and neurovascular function. (a)** Randall-Sellito tail pressure test. Mechanical withdrawal thresholds to nociceptive tail pressure. **(b)** Endothelium-dependent vasodilation. Maximal percentage of vasodilation from baseline in response to iontophoretic delivery of Ach. **(c)** Cutaneous typical laser Doppler blood flow response during a progressive increase of pressure in a control mice (black) and in a diabetic mice (red). P: start of pressure, a.u.: arbitrary units. **(d)** Pressure-induced vasodilation. Maximal percentage of vasodilation from baseline during localpressure application. (n = 10 in each group,non-parametric Kruskal-Wallis test followed by Dunn’s *post-hoc* test, *P < 0.05, **P < 0.01, ***P < 0.001)Ach: Acetylcholine, LDF:Laser Doppler Flowmetry.

After 8 weeks of diabetes, the tail pressure Randall-Sellito test evidenced a significant mechanical hypoalgesia in untreated diabetic mice (Figure 2a). The mechanical nociceptive threshold was enhanced in untreated diabetic mice (192.2 ± 19.22 g versus 126.5 ± 7.4 g in controls, p < 0.05), but was restored in the candesartan-treated diabetic group to the level of its respective control group (157.1 ± 6.1 g versus 141.6 ± 8 g, NS).

### Assessment of endothelium-independent and -dependent responses

#### Assessment of endothelium-dependent response

In the untreated control group, iontophoretic delivery of ACh increased LDF, corresponding to a maximal vasodilation of 33.3 ± 7.3%. Diabetes markedly blunted endothelium-dependent vasodilation to 8.6 ± 2.5%, (p < 0.01 versus Control). Treatment of the diabetic mice with candesartan significantly improved the ACh-induced vasodilation(31.3 ± 5.8%, p < 0.05 versus Diabetic) and restored to the level of the non diabetic control groups (NS versus Control) (Figure 2b).

#### Assessment of endothelium-independent response

In all groups, LDF increased in response to iontophoretic delivery of sodium nitroprusside, and the endothelium-independent vasodilatory response was similar in the four groups of mice (data not shown).

### Assessment of pressure-induced vasodilation

In the control mice, the progressive increase in local pressure application elicited the normal biphasic PIV response, i.e. a progressive increase in LDF peaking at 0.4 kPa then reversing to a progressive decrease, with a maximal vasodilatory response of 34.7 ± 8.4% (Figure 2c). In contrast, the normal PIV response was abolished in the untreated diabetic mice, resulting in a negative change of LDF from baseline at 0.4 kPa (−16.7 ± 6.5%, p < 0.001 versus Control) (Figure 2c). Treatment with candesartan had no effect in control mice (34.1 ± 8%), and fully restored a normal PIV response in diabetic mice (27.73 ± 10.5%, p < 0.05 versus Diabetic, NS versus Control) (Figure 2d).

### Effects of candesartan on pressure-induced ulcer formation

**Figure 3:**
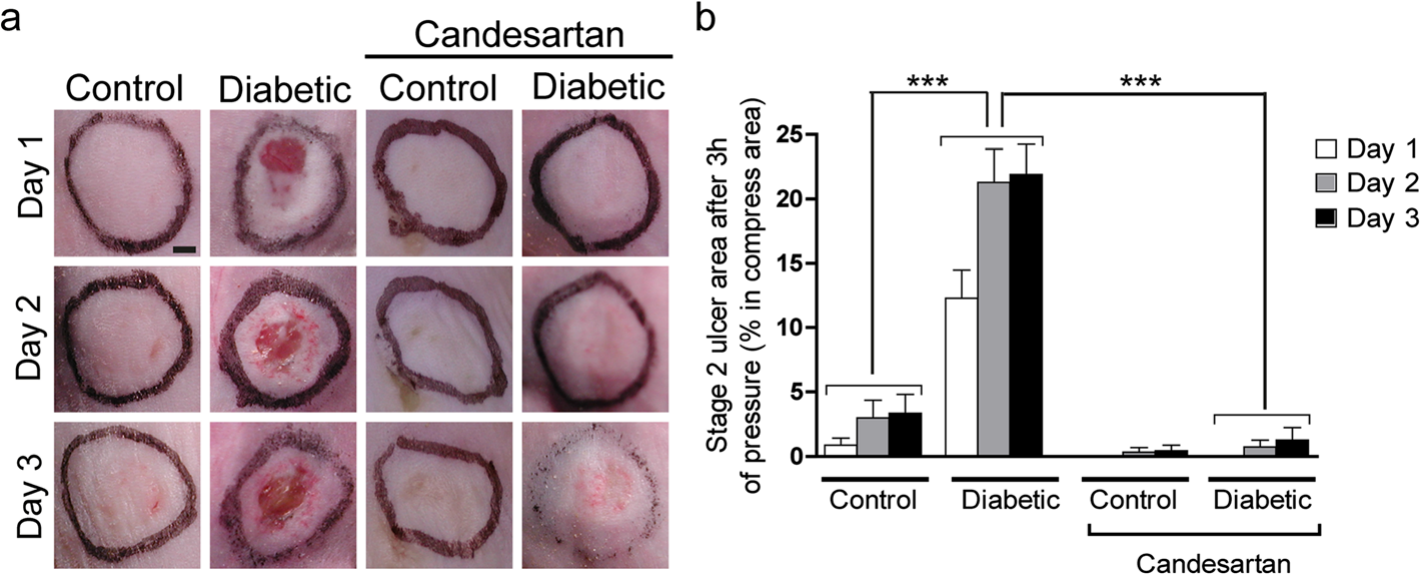
**Effect of candesartan on cutaneous macroscopic findings following 3 hours of pressure. (a)** Representative photographs of skin compressed areas, one day and three days after pressure release. Scale bar = 2 mm **(b)** Time course of macroscopic stage 2 ulcer area. (n = 20 in each group, non-parametric Kruskal-Wallis test followed by Dunn’s *post-hoc* test, ***P < 0.001).

**Figure 4:**
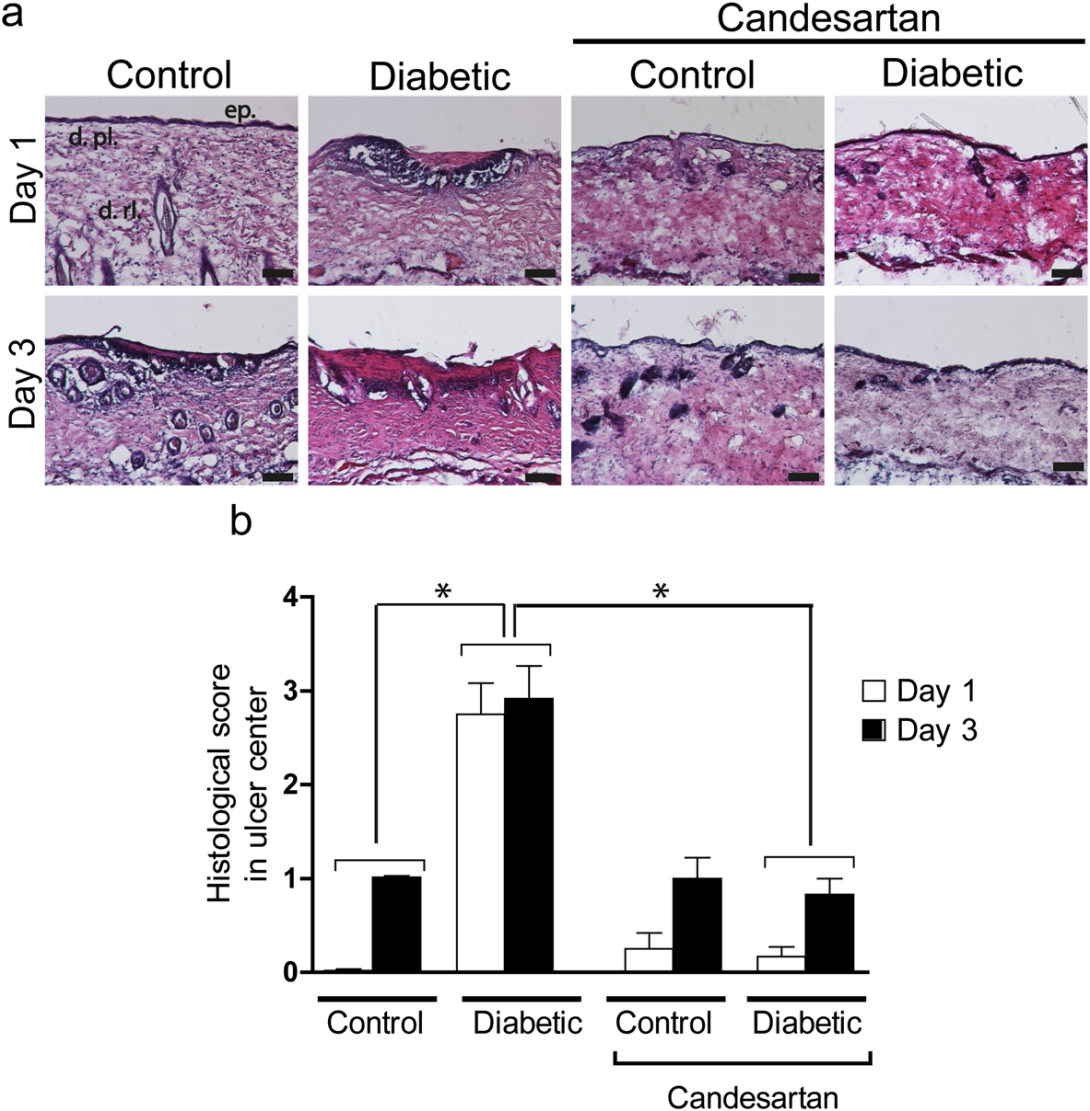
**Effect of candesartan on histological findings following 3 hours of pressure. (a)** Representative photographs of central histological skin compressed section, one day and three days after pressure release. Ischemic skin lesions were removed 24 h and 72 h after pressure release and stained with hematoxylin and eosin. ep: epidermis, d.pl: dermis-papillary layer, d.rl: dermis-reticular layer. Scale bar = 100 μm. **(b)** Central histological pressure score (n = 6 in each group, *P < 0.05).

Exposure to pressure of the back skin in control mice elicited modest and inconstant skin lesions (Figure 3a, b). After three days, the mean lesion surface was 3.3 ± 1.5% of the compressed area. Histological examination of the lesion center showed a thickening of the epithelium with the remaining presence of three skin layers, yielding a score of 1 ± 0.02. The diabetic mice developed larger and much severe lesions: the mean ulcer area was 21.9 ± 2.3%, and histological examination mainly showed epidermal and dermal necrotic defects with a score of 2.9 ± 0.35 (Figure 4a, b). Treatment with candesartan completely restored the ability of the diabetic skin to resist to pressure-induced ischemia/reperfusion injury, as the size and the depth of the lesions were similar to that observed in the non-diabetic controls.

## Discussion

The main findings of our study are that the ARB candesartan, administered for two weeks in mice with diabetes of six weeks duration with established neuropathy (1) restored endothelium-dependent relaxation, improved functional nociceptive hypoalgesia, and fully restored a normal PIV response, and (2) restored the ability of the diabetes mice skin to resist to pressure-induced ischemia as efficiently as in the control mice.

### Effect of candesartan on endothelial function

The beneficial effect of candesartan on endothelial function in diabetes has been documented by more than two decades and is not a novel finding. The understanding that activation of the RAS during diabetes increase oxidative stress that in turn converts nitric oxide synthase from an NO-producing enzyme to an enzyme that functions as a NADPH oxidase, and generates superoxide and hydrogen peroxide, has been widely documented. This process, termed NOS uncoupling, resulting in less NO and more superoxide generation, has been established as a central underlying cause of endothelial dysfunction found in atherosclerosis, hypertension, diabetes and ischemia/reperfusion [18,19]. Evidence has accumulated, both in experimental models [20-23] and clinical studies [24-29], in a broad variety of vascular beds and organs, that reducing the RAS activation, either with ACE inhibitors or ARBs, reduces inflammation, improves endothelial dysfunction caused by hypertension and/or diabetes, recouples eNOS and restores NO availability. Although not documented in our study, it makes thus little doubt that the beneficial effect of candesartan on endothelial function was linked to the improvement of the well-established effect of ARBs on diabetes-induced imbalance of NO and ROS production in the skin vasculature. Accordingly, the effects of candesartan herein reported likely reflect a class effect. However, some heterogeneity among the various sartans has been reported [30] and further studies are required to confirm that our results reflect a generic property of AT1 blockade and are not restricted to candesartan.

### Effect of candesartan on sensory neuropathy

Like diabetic patients, mice with streptozotocin-induced diabetes develop peripheral diabetic neuropathy with alterations in nociceptive thermal and mechanical thresholds [31] corresponding to a deterioration of small sensory fibers (C and Aδ fibers); the first fibers affected in diabetic neuropathy [32]. In Swiss mice, thermal hypoalgesia develops after only two weeks of streptozotocin-induced diabetes and deficit of intraepidermal nerve fiber density is present after 4 weeks [33]. In our study, Candesartan restored a normal nociceptive threshold in diabetic mice with 8 weeks of diabetes in accordance with reported evidence supporting a beneficial effect of opposing angiotensin II in diabetic neuropathy. Experimental [12] and clinical data [14,34] suggest that ACEIs have beneficial effects on diabetic neuropathy. Directly opposing angiotensin II with AT1 blockade has been similarly reported to ameliorates intraepidermal fiber density in type 2 diabetic rat [13], and to improve sciatic epineurial arteriole reactivity, preventing neuronal ischemia [12] in streptozotocin-diabetic rats. Although the mechanisms of the protective effect of RAS blockade on diabetic neuropathy remain unclear, both the improvement of vascular function and the reduction of oxidative stress and inflammation have been proposed to be involved [12].

Candesartan restored a normal PIV and the ability of the diabetic mice skin to resist to pressure-induced ischemia. As anticipated, the restoration of both endothelial function and nociceptive perception restored a normal PIV response in our model. This was associated with a fully restored capacity of the diabetic mice to resist of pressure-induced skin ulcer. The question however remains as to whether the later is a direct consequence of the former.

Fromy et al. [5] have established a direct causative link between PIV and the skin ability to resist to pressure induced ulceration, by demonstrating that genetically modified mice lacking Asic3, in contrast to their wild type controls, have a defective PIV and develop skin ulcer after exposure to pressure. Because the pressure applied to the skin was orders of magnitude higher than the threshold pressure value that elicits the maximal vasodilatory response, skin perfusion was completely suppressed under the magnets, whether PIV was present or not. The authors showed that, skin blood flow rapidly recovered after pressure release in the control mice, but not in the Asic3^−/−^ mice. The proposed explanation is that Asic3 is not only a mechanosensitive channel, but is also activated by protons and lactates. The rapid reperfusion and reoxygenation of the skin would thus reflect the vasodilatory response to ischemia-induced tissular acidification, lacking in Asic3^−/−^ mice. PIV thus can be viewed as a surrogate functional marker, allowing a simple and non-invasive assessment of the integrity of a physiological mechanism involving post-ischemia acidification-induced hyperemia, a necessary condition to allow the normal skin to resist to pressure ulcer. It is thus tempting to speculate that in our study, the restoration of a normal PIV by candesartan was necessary and sufficient for allowing the skin of the diabetic mice to resist to pressure ulcer, but this cannot be concluded with certainty. Indeed, in the very same experimental model, we have previously shown that treatment with recombining human erythropoietin (rhEPO) restored skin innervation density and C-fiber nociception and fully prevented pressure ulcer development in diabetic mice [2]. However, rhEPO had no effect on endothelial dysfunction and failed to restore a normal PIV. It is therefore possible that the protection against pressure ulcer by candesartan, like rhEPO, targets a mechanism of protection against ischemia-reperfusion, bypassing its beneficial effect on PIV.

Importantly, whereas ACE inhibitors decrease Ang II circulating levels, blockade of the AT1 receptor has the opposite effect. By blunting the AT1-mediated negative feedback of angiotensin II on renin release, it results in increased circulating levels of angiotensin II and its catabolites, angiotensin III and angiotensin IV, leading to increased stimulation of unopposed AT2 and AT4 (IRAP) receptors [35]. A protective effect of ARBs in cerebral ischemia has been largely documented, and has been shown to be dependent upon non-AT1 receptors [36-43], whereas ACE inhibition was deleterious or ineffective [37,39,43,44]. Therefore, a central question raised by our present findings is whether the skin protective effect of candesartan in the present study is solely the consequence of reducing the stimulation of AT1 by angiotensin II (AngII), or if it involves ARB induced non-AT1 mechanisms. Further studies are required to elucidate the mechanism of the protective effect of candesartan against pressure-induced skin ischemia, and a direct head to head comparison with the effect of ACE inhibition clearly appears as the first step.

As recalled above, ACE inhibitors share the protective effect of ARBs against diabetes induced endothelial dysfunction and peripheral neuropathy, and would thus be expected to be as effective to restore PIV. However it is possible that, despite restoring a normal PIV, ACE inhibitors fail to restore the diabetes skin ability to resist to ischemia, if this later effect depends upon non-AT1 receptors activation. Indeed, a recent study by Margolis et al. [45] supports this hypothesis. In this retrospective cohort study conducted in 40,000 individuals with diabetes treated with either ACE inhibitors or ARBs, the risk of foot ulcer, and the risk of lower limb amputation for those with peripheral arterial disease were both twice higher for those on ACE inhibitors. Although the intrinsic limitations inherent to population based retrospective cohort studies preclude a definitive conclusion, this impressive difference in foot ulcer risk makes the hypothesis worth considering.

Whether the restoration of a normal PIV is involved or not, the most salient finding of our study is that in the murine model of streptozotocin-induced diabetes, a rescue treatment with a non-hypotensive dose of candesartan administered for 2 weeks after 6 weeks of diabetes was able to prevent the development of deep skin ulcers in response to an imposed pressure insult, a finding that may open to important therapeutic perspectives. Certainly, further preclinical studies are mandatory to replicate our findings in other models of experimental diabetes, but such independent confirmation of our results would strongly invite to consider implementing a clinical trial to evaluate the protective effect against foot ulcerations of early ARB treatment in normotensive non-albuminuric diabetes patients. Indeed, the incidence of diabetes and its complications is increasing worldwide, and the lifetime prevalence of foot ulcers is estimated to 10 to 20% [46,47] with devastating consequences for the patients and a considerable economic burden for the health care systems, whereas ARBs treatment are relatively inexpensive and have been largely demonstrated to be safe, well tolerated and beneficial in diabetic patients.

## Abbreviations

ACE: Angiotensin converting enzyme
ACEI: Angiotensin converting enzyme inhibitor
Ach: Acetylcholin
ARB: Angiotensin receptor blocke
AT1R: Angiotensin II type 1 receptor
CGRP: calcitonin gene-related peptide
LDF: Laser Doppler flowmetry
NO: Nitric oxide
PIV: Pressure-induced vasodilation
RAS: Renin-angiotensin system
SABP: Systolic arterial blood pressure
SNP: Sodium nitroprusside
STZ: Streptozotocin
